# 17-DMAG, an HSP90 Inhibitor, Ameliorates Multiple Organ Dysfunction Syndrome via Induction of HSP70 in Endotoxemic Rats

**DOI:** 10.1371/journal.pone.0155583

**Published:** 2016-05-25

**Authors:** Yi-Li Wang, Hsin-Hsueh Shen, Pao-Yun Cheng, Yen-Ju Chu, Hwong-Ru Hwang, Kwok-Keung Lam, Yen-Mei Lee

**Affiliations:** 1 Graduate Institute of Life Sciences, National Defense Medical Center, Taipei, Taiwan; 2 Department of Pharmacology, National Defense Medical Center, Taipei, Taiwan; 3 Department of Physiology & Biophysics, National Defense Medical Center, Taipei, Taiwan; 4 Division of Cardiology, Department of Medicine, Kaohsiung Veterans General Hospital, Kaohsiung, Taiwan; 5 Department of Pharmacology, Taipei Medical University, Taipei, Taiwan; 6 Department of Anesthesiology, Catholic Mercy Hospital, Hsinchu, Taiwan; National Institutes of Health, UNITED STATES

## Abstract

Sepsis is a systemic inflammatory disorder, accompanied with elevated oxidative stress, leading to multiple organ dysfunction syndrome (MODS), and disseminated intravascular coagulation. 17-Dimethylaminoethylamino- 17-demethoxygeldanamycin (17-DMAG), a heat shock protein (HSP) 90 inhibitor, has been reported to possess anti-inflammatory effects. In this study, the beneficial effects of 17-DMAG on lipopolysaccharide (LPS) induced MODS and DIC was evaluated in anesthetized rats. 17-DMAG (5 mg/kg, i.p.) was significantly increased survival rate, and prevented hypotension in LPS (30 mg/kg i.v. infused for 4 h) induced endotoxemia. The elevated levels of alanine aminotransferase (ALT), creatine phosphokinase (CPK), lactate dehydrogenase, creatinine, nitric oxide (NO) metabolites, IL-6, and TNF-α in LPS-exposed rat plasma were significantly reduced by 17-DMAG. Moreover, 17-DMAG suppressed LPS-induced superoxide anion production and caspase 3 activation in heart tissues. LPS induced the prolongation of prothrombin time, and a pronounced decrease in platelet count, which were improved by 17-DMAG. 17-DMAG markedly induced HSP70 and heme oxygenase (HO)-1, and suppressed inducible nitric oxide synthase (iNOS) and phosphorylated NF-κB p65 protein expression in organs 6 h after LPS initiation. Pretreatment with high dose of quercetin (300 mg/kg, i.p.), as an HSP70 inhibitor, reversed the beneficial effects of 17-DMAG on survival rate, plasma levels of ALT, CPK, creatinine, IL-6, and NO metabolites, iNOS induction, and caspase-3 activation in LPS-treated rats. In conclusion, 17-DMAG possesses the anti-inflammatory and antioxidant effects that were proved through LPS-induced acute inflammation, which is associated with induction of HSP70 and HO-1, leading to prevent MODS in sepsis.

## Introduction

Sepsis is the clinical syndrome of a systemic inflammatory response that complicates severe infection. Gram-negative bacteria, like *Escherichia coli* (*E*. *coli)* and *Candida* sp., are the most common infecting bacteria in sepsis. Lipopolysaccharide (LPS), a component of the outer membrane of G(-)-bacterial cell wall, would bind to the toll-like receptor 4 on the membrane of macrophage or neutrophil, leading to activation of NF-κB pathway, which then induces the pro-inflammatory factors, such as TNF-α and IL-6, and inducible nitric oxide synthase (iNOS) expression and NO overproduction [1,2]. The activation of macrophages and neutrophils release a large number of superoxide anions and other oxidants in infected cells and organs. Those factors would induce the systemic inflammatory response syndrome, and multiple organ dysfunction syndrome (MODS) [3].

Disseminated intravascular coagulation (DIC) is a common complication of sepsis [4]. The release of cytokines by endotoxins triggers the expression of tissue factor on endothelial cells and monocytes, which initiates activation of the coagulation cascade, leading to decreased blood perfusion, inadequate oxygenation and multiple organ dysfunction or failure. In late-stage of DIC, due to consumption and exhaustion of coagulation factors and platelets, incidence of bleeding increases [5].

Heat shock protein 70 (HSP70) is one of the co-chaperone proteins exist in all living organisms. HSP70 can be reported to re-fold misfolding or unfolding proteins in cancer and other stress-related diseases [6]. HSP70 protects aged mice against insults from cecal ligation and puncture-induced and bacteria-infected sepsis by its anti-inflammatory effects [7]. Prophylactic intravenous injection of HSP70 significantly reduces mortality rates and inflammatory responses in lipoteichoic acid-induced sepsis, and attenuates reactive oxygen species (ROS) production in neutrophils [8]. These evidences demonstrate that HSP70 plays an important role in maintaining cellular homeostasis to defend organs from bacterial infection and acute inflammation-evoked damages.

HSP90 is one another chaperone protein to refold the abnormal proteins. HSP90 has been reported to be up-regulated in various diseases, including cancer [9]. Geldanamycin, an HSP90 inhibitor, can bind to ATP-binding pocket of the HSP90 dimer, resulting in the attenuation of HSP90 activation [10], and shows promise to treat cancers [9]. Recently, the HSP90 inhibitors were reported to possess anti-inflammatory effects, associated with activation of heat shock factor (HSF)-1, leading to induction of HSP70 production, but no significant effect on HSP90 protein expression [11]. A geldanamycin analog, 17-Dimethylamino-ethylamino-17-demethoxygeldanamycin (17-DMAG), an HSP90 inhibitor, is characterized by a more water-soluble and less hepatotoxic [12]. 17-DMAG is found to inhibit oxidative stress in animal models of atherosclerosis and kidney ischemia-reperfusion injury [13,14]. Therefore, in this study, we evaluated the protective effects of 17-DMAG on MODS in an animal model of LPS induced sepsis, and explored the possible mechanisms. We found that 17-DMAG improved MODS and survival rate, accompanied by suppressive effects on inflammatory responses and oxidative stress during sepsis. Induction of HSP70 and HO-1 is associated in the perceived beneficial effect.

## Materials and Methods

### Chemicals

17-DMAG was purchased from InvivoGen (San Diego, CA, USA). LPS from *Escherichia coli* 0127:B8 was purchased from Sigma-Aldrich (St. Louis, MO, USA). Quercetin was purchased from Cayman Chemicals (Ann Arbor, MI, USA).

### Experimental animals

Male Wistar-Kyoto rats (275–310 g) were obtained from the BioLASCO Taiwan Co., Ltd. The animals handling was in accordance with the *Guide for the Care and Use of Laboratory Animals* published by the U.S. National Institutes of Health (NIH Publication No. 85–23, revised 1996). All animals were housed at an ambient temperature of 22 ± 1°C and humidity of 55 ±5%. The protocol was approved by the National Defense Medical Center Institutional Animal Care and Use Committee, Taipei, Taiwan (Permit Number: IACUC-15-019).

According to the methods described by Asakura et al., [15] and Kung et al., [16] experimental sepsis and DIC were induced by the sustained iv infusion of 30 mg/kg LPS diluted in 9 ml saline for a maximum of 4 h. For this, rats were anesthetized by intraperitoneal injections of urethane (0.6 g/kg) and pentobarbital (30 mg/kg). The trachea was cannulated to facilitate respiration. The left femoral artery was cannulated with a polyethylene-50 (PE-50) catheter and connected to a pressure transducer (MLT844, ADInstruments Pty Ltd, Castle Hill, NSW, Australia) for the measurement of mean arterial blood pressure (MAP) and heart rate, which were displayed on a polygraph recorder (ML 785, PowerLab, ADInstrument). The left femoral vein was cannulated for the administration of drugs. After the surgery was completed, rats were allowed to stabilize for 30–60 min, and then they were divided into six groups: (1) Sham: rats were administered a sustained intravenous infusion of 9 mL normal saline (vehicle of LPS) for 4 h via femoral vein (N = 5); (2) 17D: rats were treated with 17-DMAG (5.0 mg/kg) 20 h before 9 mL normal saline given (N = 5); (3) LPS: rats were treated with sustained i.v. infusion of 30 mg/kg LPS diluted in 9 mL saline for a maximum of 4 h via femoral vein (N = 20); (4) 17D+LPS: rats were intraperitoneally (i.p.) treated with 17-DMAG (5.0 mg/kg) 20 h before LPS (30 mg/kg, i.v. infusion for 4 h) exposure (N = 10); (5) LPS+17D: rats were given 17-DMAG (5 mg/kg i.v. infusion for 5 min) and LPS (30 mg/kg, i.v. infusion for 4 h) concurrently (Co-treatment) (N = 10); (6) 17D+Q+LPS: rats were pre-treated with 17-DMAG (5.0 mg/kg, i.p.) and quercetin (300 mg/kg, i.p.) 20 and 6 h respectively prior to LPS (30 mg/kg, i.v. infusion for 4 h) exposure (N = 10). Quercetin was used as an HSP70 inhibitor [17,18]. Hemodynamic changes, mean arterial blood pressure (MAP) and heart rate, were periodically monitored throughout the experimental period. Rats were euthanized by intravenous injection of pentobarbital (30 mg/kg), when MAP noticed below 40 mmHg. Survival or death of rat was recorded every 30 min during 6 h post LPS initiation to evaluate the survival rate in each group. Hepatic function index (alanine aminotransferase [ALT]), rhabdomyolysis marker (creatine phosphokinase [CPK]), cell toxicity index (lactate dehydrogenase [LDH]), and renal function index (creatinine [CRE]) were observed at basal and 2, 4, and 6 h after LPS initiation, which were determined by a Fuji DRI-CHEM 3030 analyzer (Fuji Photo Film, Tokyo, Japan). Six hours after saline or LPS initiation, animals were sacrificed under deep anesthesia, which was induced by administration of additional pentobarbital (15 mg/kg, iv). Hearts, aorta, lungs, and livers were collected immediately for further *ex vivo* studies.

### Plasma nitrite/nitrate determination

Aliquots (30 μL) of thawed plasma taken 6 h after LPS initiation were deproteinated with 100 ml of 95% alcohol for 30 min (4°C). Serum samples were then centrifuged for 6 min at 12,000 × *g*. The supernatant (6 μL) was injected into a collection chamber containing 5% VCl_3_. In this strongly reducing environment, both nitrate and nitrite were converted to NO. A constant stream of helium gas was used to carry the output into an NO analyzer (Sievers 280NOA; Sievers Instruments, Boulder, CO, USA), where the NO reacts with ozone (O_3_), resulting in the emission of light. Light emission is proportional to the NO formed. Standard amounts of sodium nitrate (Sigma-Aldrich, St. Louis, MO, USA) were used for calibration.

### Plasma TNF-α and IL-6 determination by ELISA

The plasma levels of TNF-α and IL-6 were detected 2 and 6 h after LPS initiation, respectively, and determined using Bio-Plex Pro rat cytokines assay kit (Bio-Rad Laboratories, USA) according to the manufacturer’s instructions.

### Superoxide anions determination

The method used to determine superoxide anion levels was as described previously [19]. In brief, myocardium samples (3 × 3 mm) were taken from left ventricle 6 h after LPS initiation, and incubated in 95% O_2_/5% CO_2_ oxygenated modified Kreb’s/HEPES solution (37°C) for 30 min. Then, the myocardium samples were put into a 96-well plate in which every well was filled with 200 μL of modified Kreb’s/HEPES solution and placed in a luminescence measurement system (Hidex, Microplate Luminometer, Turku, Finland). This system injected 125 μM lucigenin (final volume of 250 μL) onto the tissues to interact with superoxide anions. Counts were obtained at 30 s intervals at room temperature. After recording was complete, all samples were dried in a drying cabinet for 2 weeks. These results were expressed as counts per second (cps) per milligram dry weight of tissues.

### Determination of reduced glutathione

The levels of reduced glutathione (GSH) in the myocardium were determined 6 h after LPS initiation using a GSH detection kit (Enzo Life Sciences, Farmingdale, NY, USA) according to the manufacturer’s instructions.

### Evaluation of coagulation function

Platelet counts and prothrombin time are considered to be important parameters to determine the changes of coagulation in endotoxemic rats [20]. Six hours after LPS initiation, platelet counts were performed by an automatic counter hematology analyzer for animals (Sysmex KX-21, Block Scientific Co., USA) within 10 min after collecting whole blood. Prothrombin time was determined by Fibrometer (BBL, Fibrosystem, Utech Products Co., USA).

### Determination of caspase 3

The caspase 3 activity in ventricular myocardium was determined 6 h after LPS initiation using Caspase-3 colorimetric detection kit (Enzo Life Sciences, Farmingdale, NY, USA).

### Western blot analysis

The hearts, lungs and livers of rats were isolated 6 h after LPS initiation and were immediately frozen in liquid nitrogen and stored at -80°C until processed. Detection of protein expression by Western blotting was performed as described previously [21]. The primary antibodies used in this experiment were mouse monoclonal anti-iNOS (1:1000; BD, USA), monoclonal anti-HO-1 (1:2000; Santa Cruz, USA), monoclonal anti-HSP70 (1:1000; Assay Designs, USA), monoclonal anti-plasminogen activator inhibitor-1 (PAI-1) (1:10000; BD, USA), monoclonal anti-β-actin (1:5000; Cell Signaling, USA), monoclonal anti-α-actin (1:2000; Sigma, USA) and rabbit polyclonal phosphorylated NF-κB p65 (pp65) antibody (1:2000; Millipore, USA).

### Histopathology

Lung tissues were dissected out after sacrifice of the rats, fixed in 10% formaldehyde, dehydrated and embedded in paraffin wax for histological studies. From the blocks, 4 μm sections were then stained with hematoxylin and eosin (H & E), mounted in DPX and examined under a microscope with original magnification of 400x to detect histopathological evidence of lung inflammation.

### Statistical analysis

The data are expressed as means±SEM. Statistical analyses were performed using one-way ANOVA followed by the Newman—Keuls post-hoc comparison test. Log-rank test was used to compare the survival rate among groups. P<0.05 was considered as statistically significant.

## Results

### 17-DMAG prevented hypotension caused by LPS

**Fig 1:**
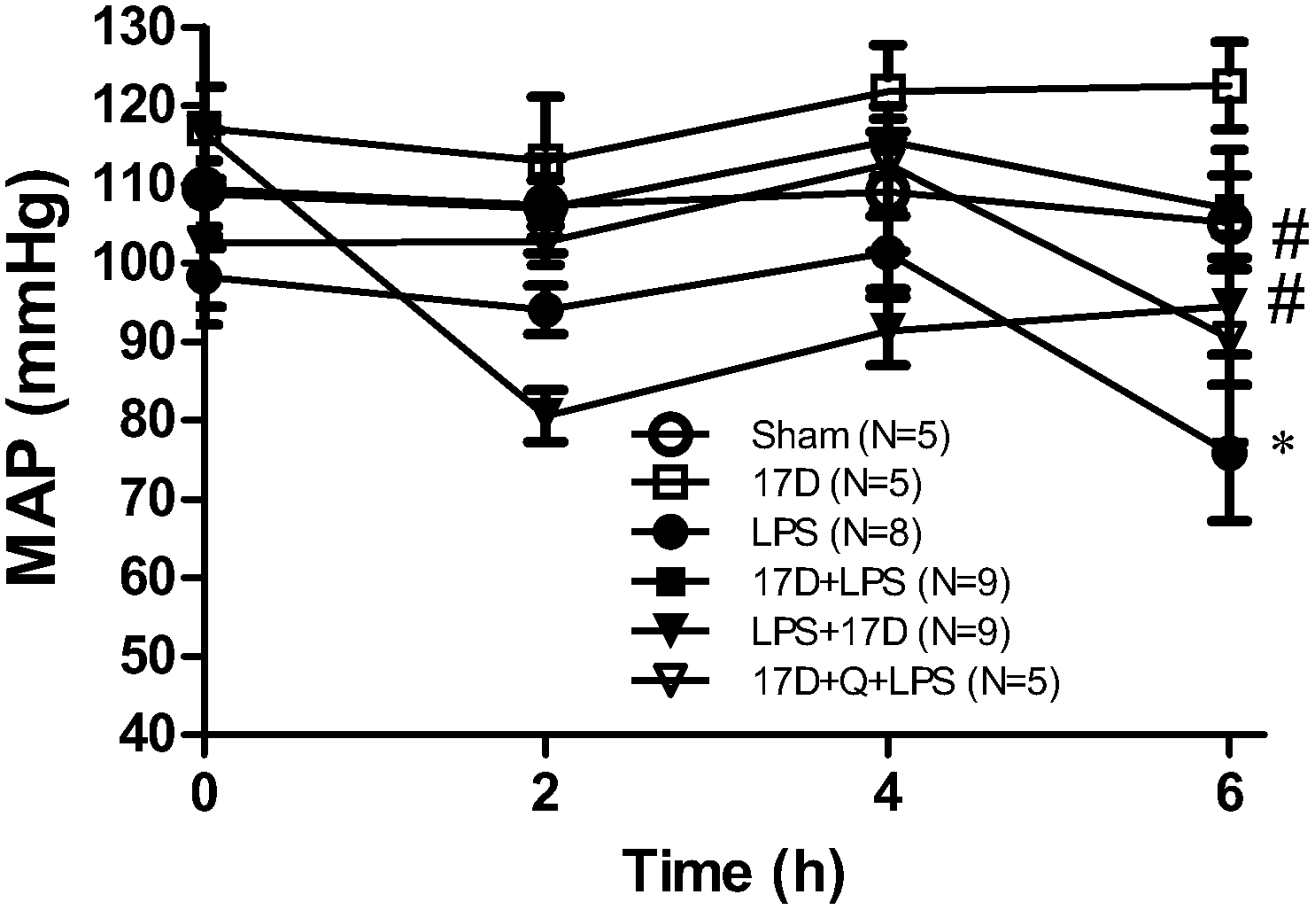
Effects of 17-DMAG (17D) on mean arterial blood pressure (MAP) in LPS induced endotoxemic rats. 17-DMAG (17D; 5 mg/kg) was given 20 h or 0 min prior to LPS initiation (30 mg/kg/4 h). Quercetin (Q; 300 mg/kg, i.p.), an HSP70 inhibitor, was given 6 h prior to LPS initiation. Values are expressed as mean±SEM. Animal number in each group is shown in the parenthesis. *P < 0.05 vs. sham group, ^#^ P < 0.05 vs. LPS group.

Six hours after LPS exposure, mean arterial blood pressure (MAP) was found to be significantly lowered than that of sham group (P< 0.05). Pretreatment and co-treatment with 17-DMAG prevented the hypotensive effect caused by LPS. Quercetin did not significantly reverse the effect of 17-DMAG on MAP (Fig 1).

### 17-DMAG prevented MODS in endotoxemic rats

**Fig 2:**
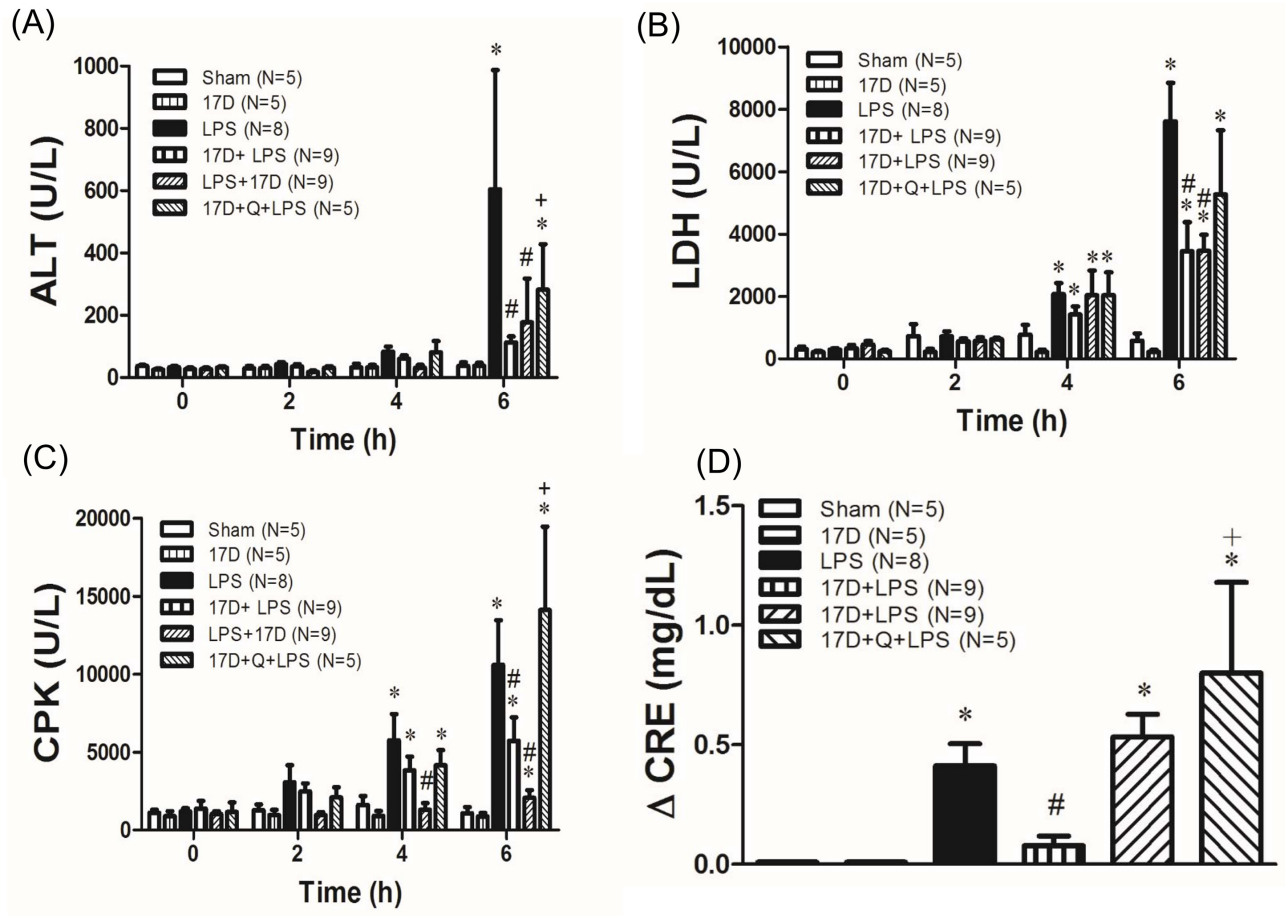
Effects of 17-DMAG (17D) on alanine aminotransferase (ALT) (A), lactate dehydrogenase (LDH) (B), creatine phosphokinase (CPK) (C), and creatinine (CRE) (D) in rats with endotoxemia induced by LPS administration. Q: quercetin; Values are expressed as mean±SEM. Animal number in each group is shown in the parenthesis. *P < 0.05 vs. sham group; ^#^P < 0.05 vs. LPS group; ^+^P < 0.05 vs.17D+LPS group.

Two hours after LPS initiation, all of parameters, including plasma levels of ALT, CPK and LDH did not significantly increase and there is no significant difference shown among groups. At 4 hr, CPK and LDH were elevated by LPS challenge, but not ALT. Co-treatment with 17-DMAG reduced the elevation of CPK, whereas 17-DMAG did not show inhibitory effect on the elevation of plasma LDH. Six hours after LPS given, the plasma levels of ALT, LDH, CPK, and the change of CRE level (ΔCRE) were significantly increased in LPS group when compared to sham group (P<0.05). Pretreatment with 17-DMAG significantly attenuated the LPS-induced plasma ALT, LDH, CPK, and ΔCRE (P<0.05). In addition, co-treatment with 17-DMAG also significantly reduced plasma levels of ALT, LDH, and CPK (P<0.05), but not ΔCRE concentrations. Moreover, quercetin significantly reversed the inhibitory effects of 17-DMAG on ALT, CPK and ΔCRE (Fig 2).

**Fig 3:**
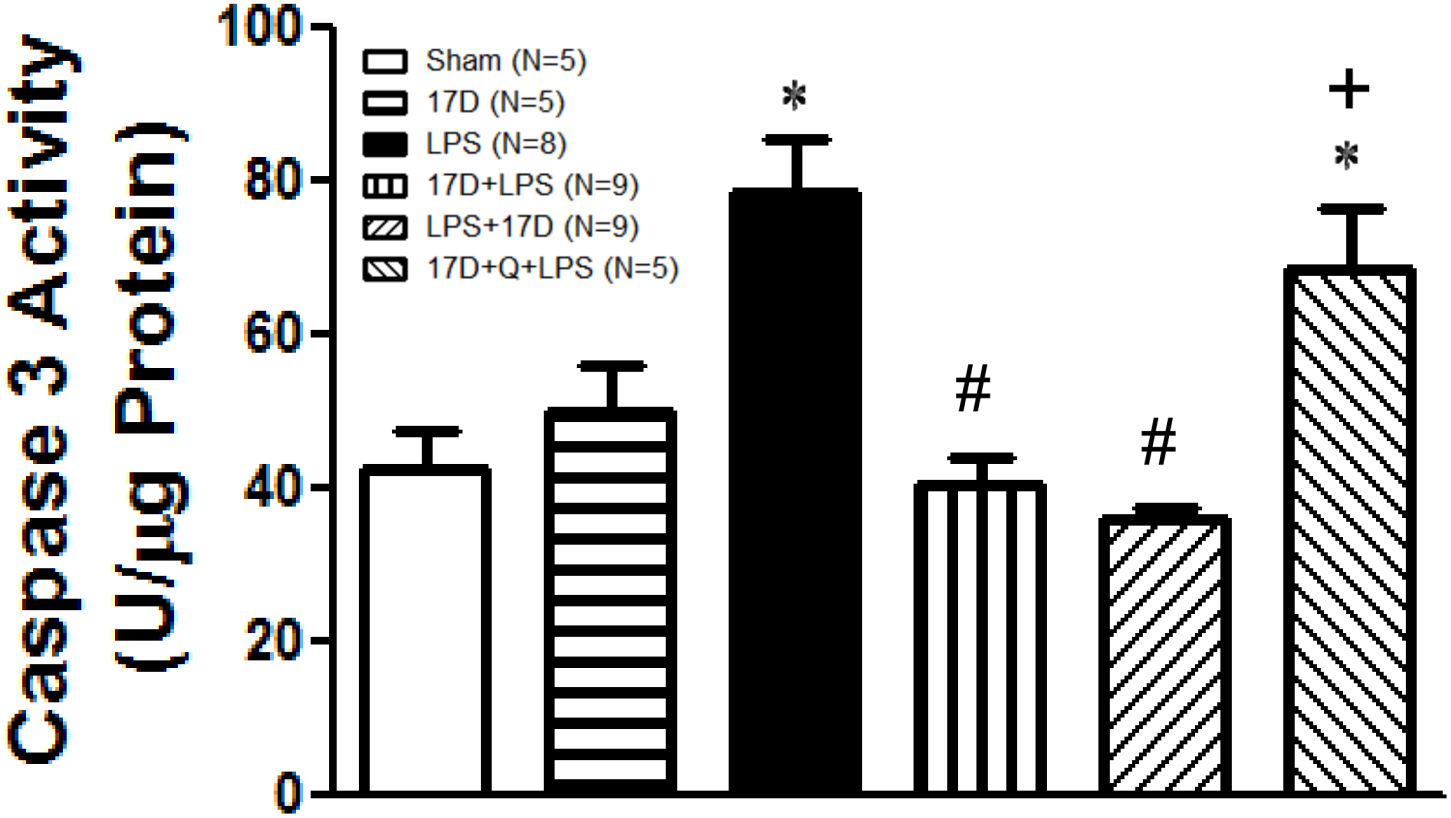
Effects of 17-DMAG (17D) on LPS-induced cardiac caspase 3 activity. Q: quercetin; Values are expressed as mean±SEM. Animal number in each group is shown in the parenthesis. *P < 0.05 vs. sham group; ^#^P < 0.05 vs. LPS group; ^+^P < 0.05 vs.17D+LPS group.

The activity of caspases 3 was determined to observe whether apoptotic process occurred in cardiomyocytes, which reflects the damage elicited by LPS leading to cardiac dysfunction [22]. Six hours after LPS initiation, caspase 3 activity was significantly elevated in LPS group when compared with sham group (P<0.05). Pretreatment and Co-treatment with 17-DMAG significantly reduced caspase 3 activity compared to LPS group (P<0.05). Pretreatment with quercetin reversed the beneficial effect of 17-DMAG on LPS-induced increase of caspase 3 activity in myocardium (P<0.05) (Fig 3).

### 17-DMAG attenuated plasma levels of NO metabolites and pro-inflammatory cytokines in endotoxemic rats

**Fig 4:**
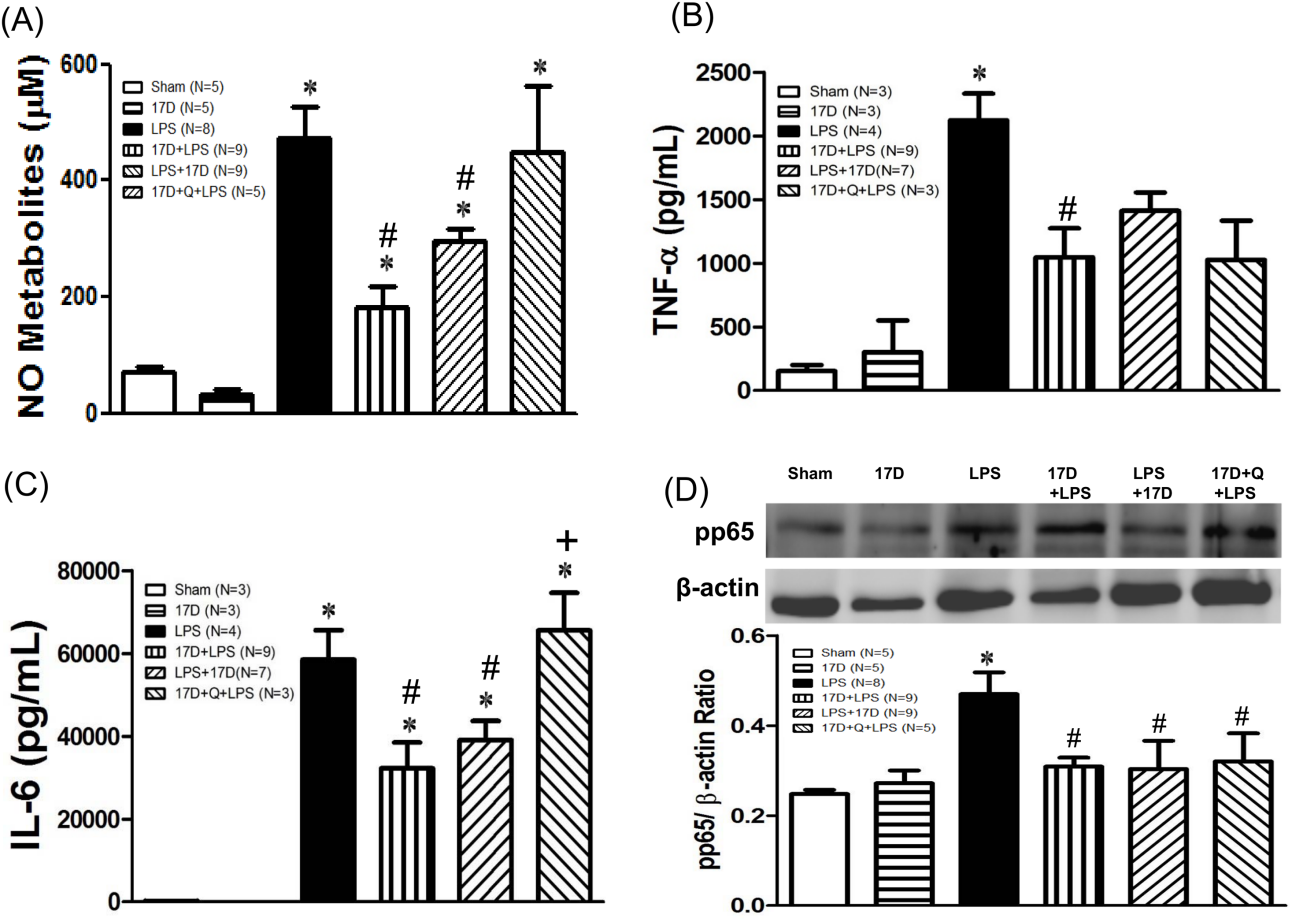
Effects of 17-DMAG (17D) on the plasma concentrations of nitric oxide (NO) metabolites (A), TNF-α (B), IL-6 (C), and phosphorylated NF-κB p65 (pp65) protein expression in lungs (D) after rats subjected to LPS administration. The level of TNF-α was detected 2 h after LPS initiation; NO metabolites, IL-6, and pp65 levels were detected after 6 h. Q: quercetin; Values are expressed as mean±SEM. Animal number in each group is shown in the parenthesis. *P < 0.05 vs. sham group; ^#^P < 0.05 vs. LPS group. ^+^P < 0.05 vs.17D+LPS group.

Plasma levels of NO metabolites, TNF-α, and IL-6 were significantly increased in LPS-exposed groups, when compared with the sham group, which were significantly reduced by 17-DMAG. Quercetin diminished the inhibitory effect of 17-DMAG on NO metabolites and IL-6, but not TNF-α levels (Fig 4A, 4B and 4C).

### 17-DMAG attenuated superoxide anion production, and increased reduced glutathione (GSH) in hearts of endotoxemic rats

**Fig 5:**
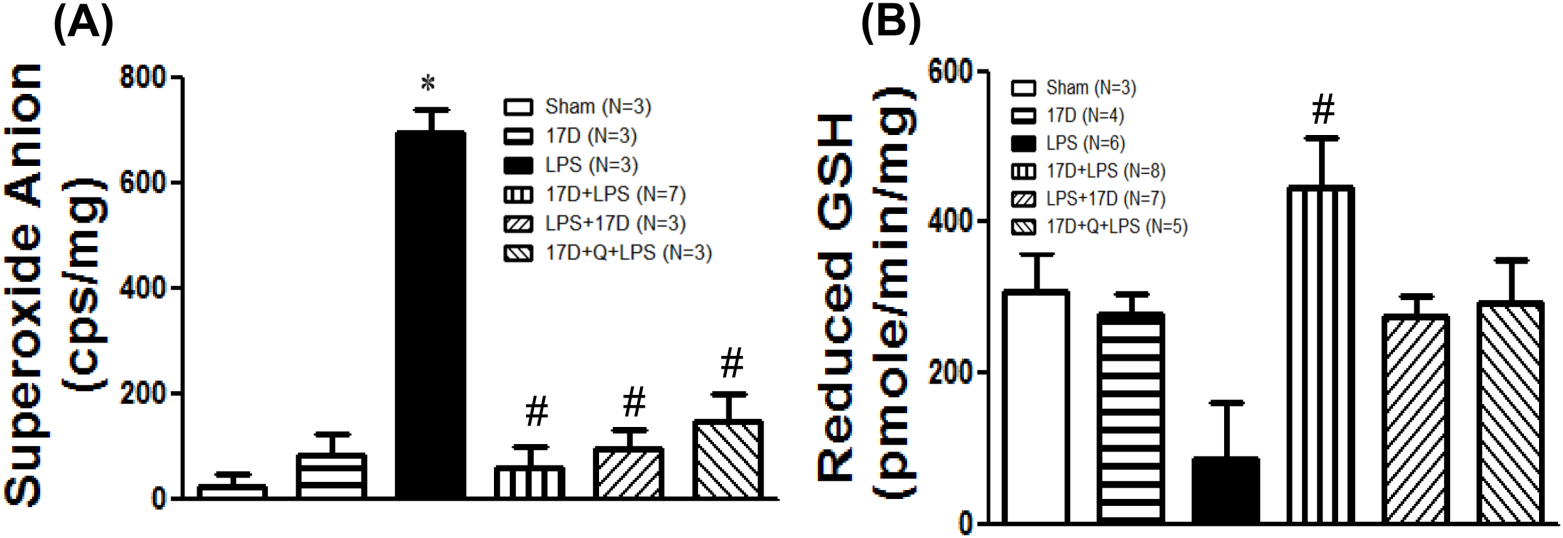
Effects of 17-DMAG (17D) on the levels of superoxide anion production (A) and reduced glutathione (B) in hearts of rats 6 h after LPS administration. Q: quercetin; Values are expressed as mean±SEM. *P < 0.05 vs. sham group; ^#^P < 0.05 vs. LPS group.

Superoxide anion production markedly increased in ventricular myocardium in the LPS group, this elevation was significantly suppressed by pretreatment and co-treatment of 17-DMAG, however, which was not affected by quercetin (Fig 5A). Pretreatment with 17-DMAG increased the levels of GSH in cardiomyocytes compared with that of LPS group (P<0.05), which did not significantly reduced by quercetin (Fig 5B).

### 17-DMAG prevented LPS-induced coagulopathy in rats

**Fig 6:**
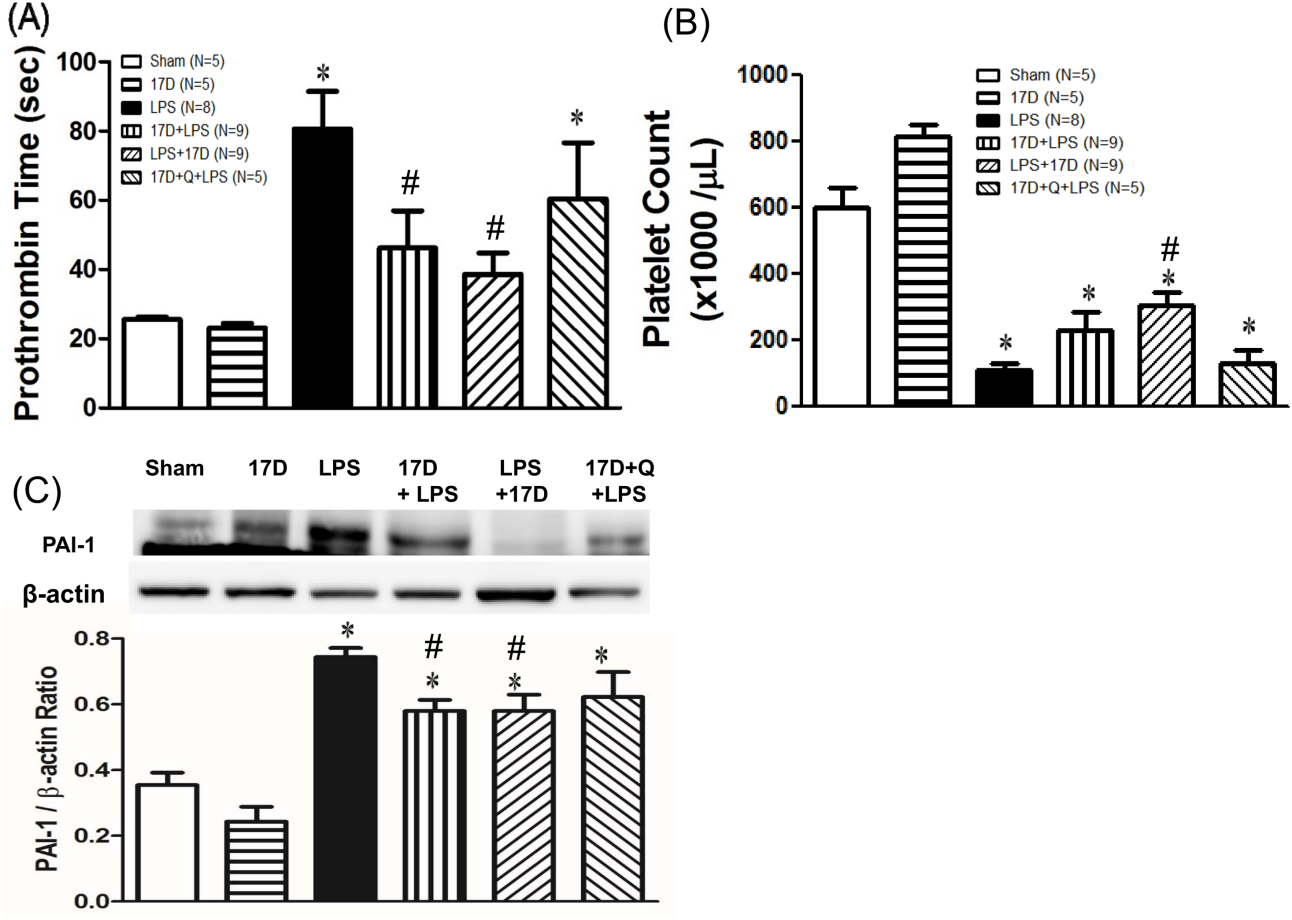
Effects of 17-DMAG (17D) on prothrombin time (A), platelet count (B) and PAI-1 protein expression in lungs (C) 6 h after LPS administration. Q: quercetin; Values are expressed as mean±SEM. Animal number in each group is shown in the parenthesis. *P < 0.05 vs. sham group; ^#^P < 0.05 vs. LPS group.

Six hours after LPS initiation, prothrombin time significantly prolonged in the LPS group, when compared with that of the sham group. Pretreatment and co-treatment with 17-DMAG pronouncedly improved the prolongation of prothrombin time. Platelet count dramatically dropped 6 h after LPS given. Co-treatment with 17-DMAG significantly ameliorated the reduction in platelet count. Furthermore, PAI-1 protein expression in lungs was up-regulated by LPS, which was lowered by pretreatment with 17-DMAG (P<0.05). The beneficial effects of 17-DMAG on prothrombin time, platelet count and PAI-1 expression were not reversed by quercetin (Fig 6).

### The effects of 17-DMAG on HSP70, iNOS, HO-1 and pp65 protein expression in hearts, lungs, and livers in endotoxemic rats

**Fig 7:**
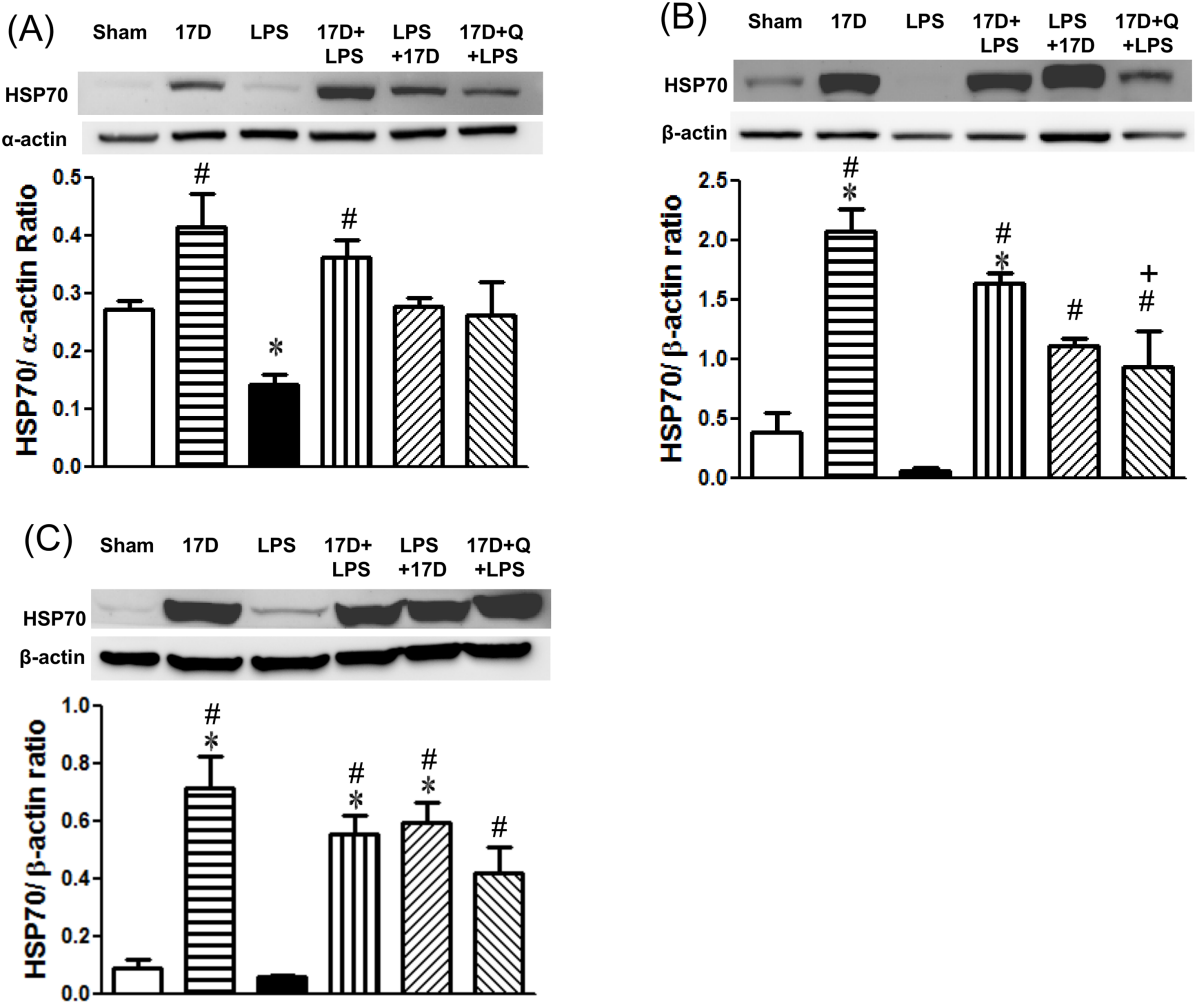
Effects of 17-DMAG (17D) on HSP70 protein expression in hearts (A), lungs (B), and livers (C) of rats 6 h after LPS administration. Q: quercetin; Values are expressed as mean±SEM. *P < 0.05 vs. sham group; ^#^P < 0.05 vs. LPS group; ^+^P < 0.05 vs.17D+LPS group.

**Fig 8:**
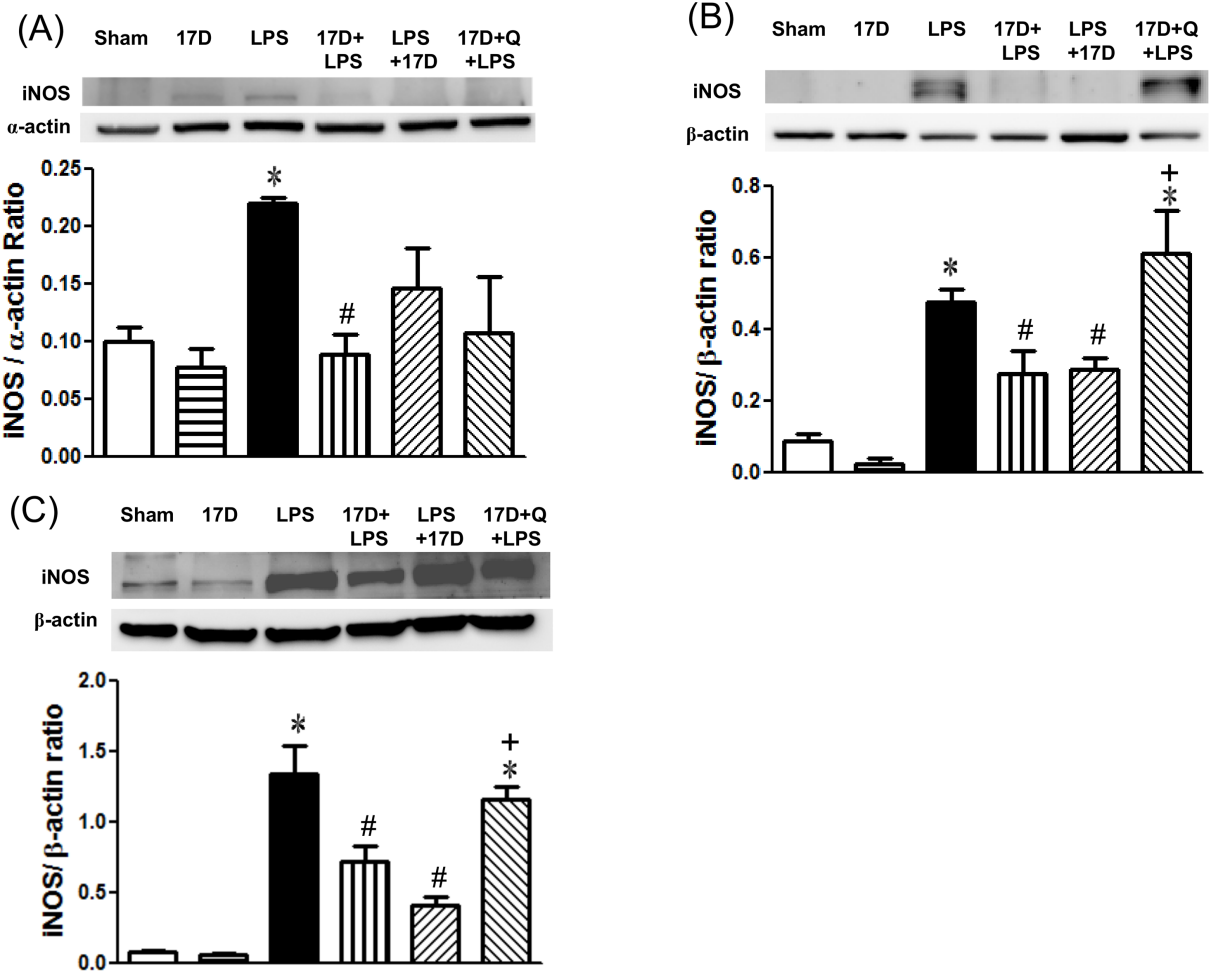
Effects of 17-DMAG (17D) on iNOS protein expression in hearts (A), lungs (B), and livers (C) of rats 6 h after LPS administration. Q: quercetin; Values are expressed as mean±SEM. *P < 0.05 vs. sham group; ^#^P < 0.05 vs. LPS group; ^+^P < 0.05 vs.17D+LPS group.

**Fig 9:**
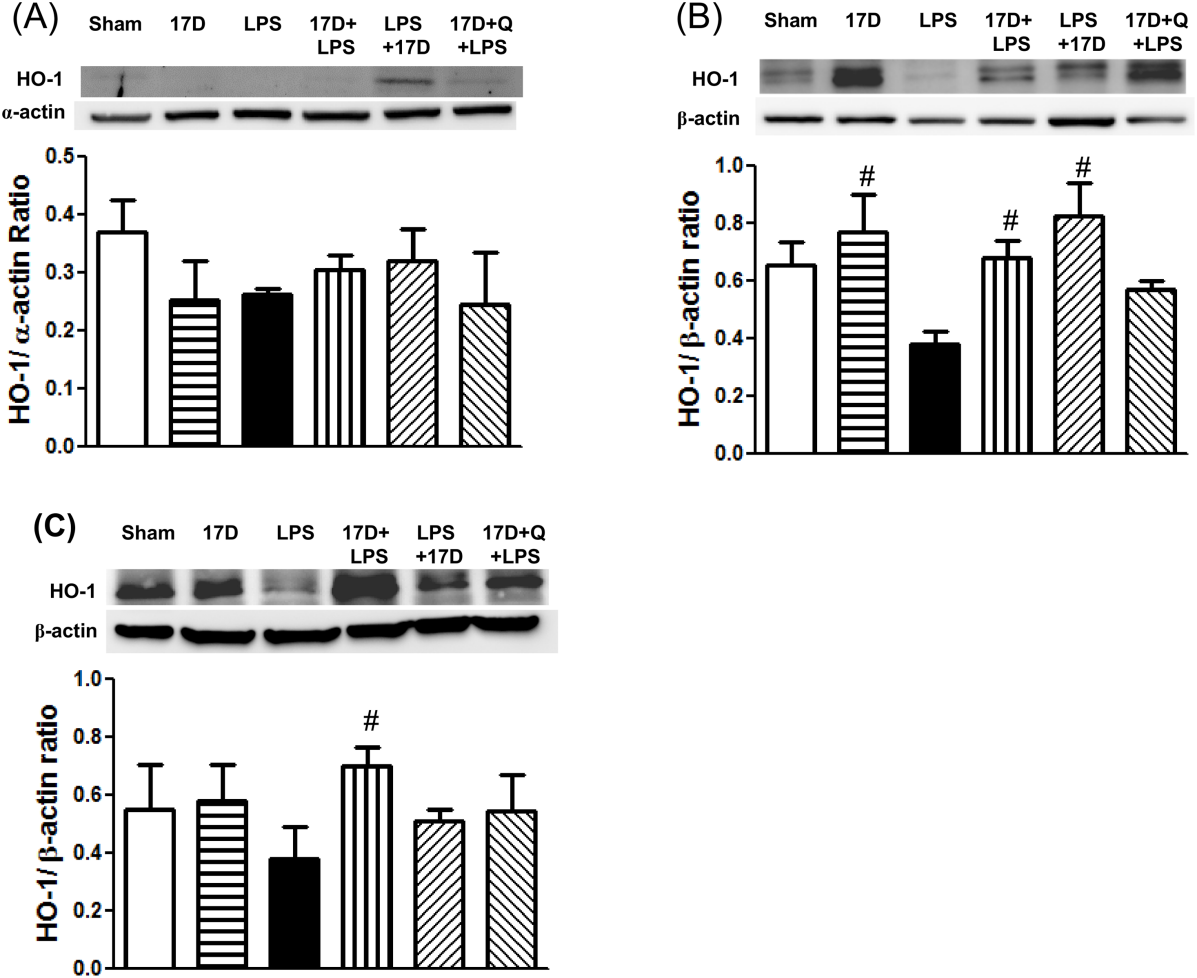
Effects of 17-DMAG (17D) on HO-1 protein expression in hearts (A), lungs (B), and livers (C) of rats 6 h after rats subjected to LPS administration. Q: quercetin; Values are expressed as mean±SEM. ^#^P < 0.05 vs. LPS group.

Pretreatment of 17-DMAG alone markedly induced HSP70 protein expression in hearts, lungs, and livers when compared with the sham group (P<0.05). Six hours after LPS given, HSP70 levels were decreased in hearts, lungs, and livers, especially in heart tissues it is significantly lowered when compared to sham group (P<0.05). Treatment with 17-DMAG dramatically elevated HSP70 levels in hearts, lungs, and livers of endotoxemic rats. HSP70 expressed in lungs was significantly suppressed by quercetin (Fig 7). Furthermore, LPS administration markedly induced iNOS expression in hearts, lungs, and livers, which were suppressed by treatment with 17-DMAG. The inhibitory effect of 17-DMAG on iNOS expression was significant reversed by quercetin in lungs and livers (P<0.05) (Fig 8). In addition, HO-1 levels in hearts, lungs, and livers slightly reduced 6 h after LPS initiation. Treatment with 17-DMAG significantly increased HO-1 protein expression in lungs and livers when compared with that of the LPS group. Pretreatment with quercetin did not significantly reduce HO-1 expression elevated by 17-DMAG in the tested organs (P>0.05) (Fig 9). Six hours after LPS initiation, NF-κB pp65 protein level markedly increased in lungs in LPS group. Pretreatment and co-treatment with 17-DMAG inhibited the LPS-induced increase in pp65. Pretreatment with quercetin did not reverse the inhibitory effect of 17-DMAG on pp65 expression (P<0.05) (Fig 4D).

### 17-DMAG reduced lung inflammation in endotoxemic rats

**Fig 10:**
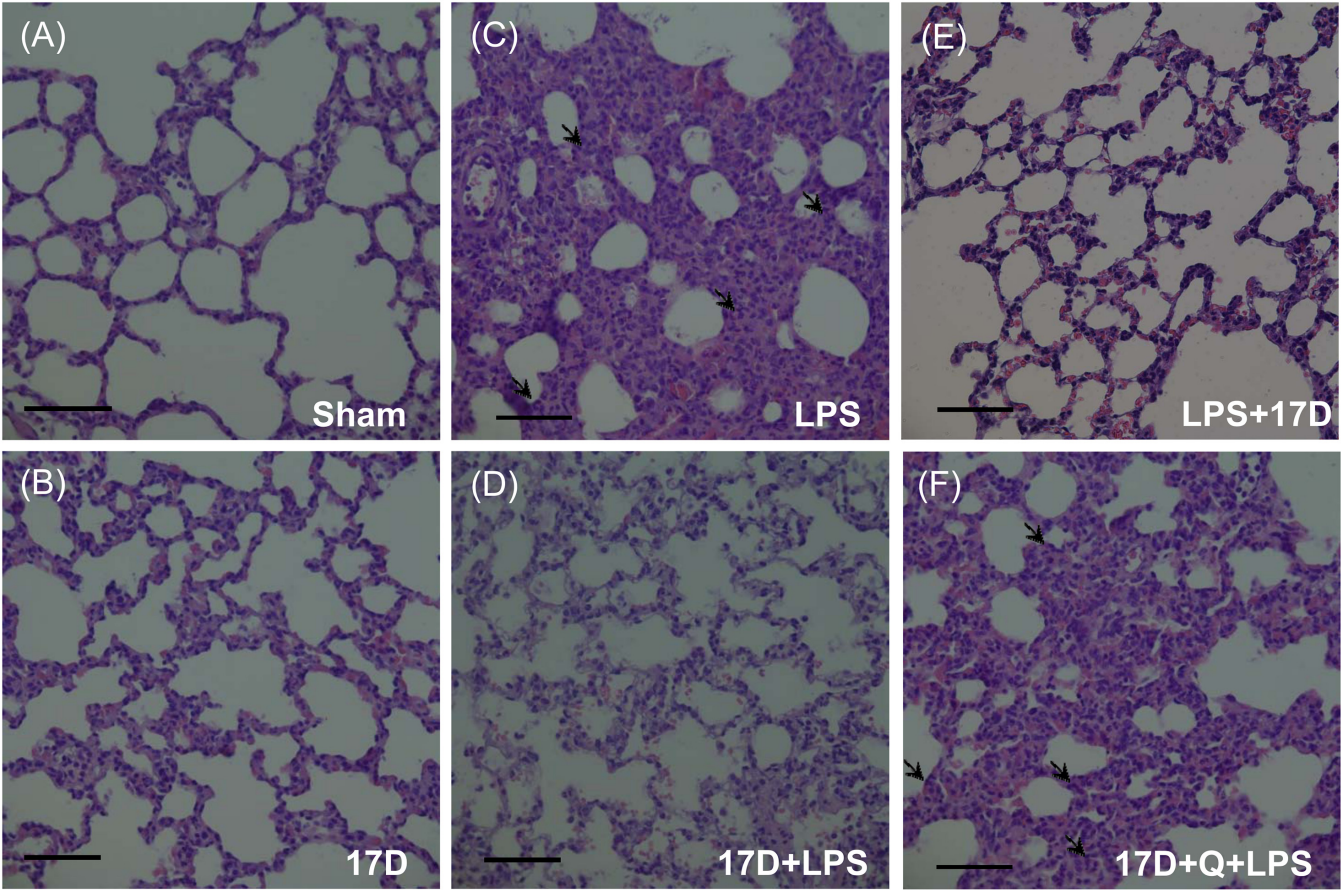
Effects of 17-DMAG (17D) on histologic studies of lung sections in the sham (A), 17D (B), LPS (C), 17D+LPS (D), LPS+17D (E), and 17D+Q+LPS groups (F). Q: quercetin; Sections were stained with hematoxylin and eosin, each X400 (original magnification). Scale bar = 50 μm. Arrows indicate infiltration of the inflammatory cells.

The histological changes of lungs were observed by H&E stain. There are no neutrophil infiltration in lungs in both sham and 17D groups. Six hours after LPS initiation, alveolar cavity pronouncedly reduced and acute inflammatory cells accumulated in thickened alveolar septa. Pretreatment and co-treatment with 17-DMAG improved these changes caused by LPS. Quercetin reversed the effects of 17-DMAG on lung damages caused by LPS (Fig 10).

### 17-DMAG improved survival rate in endotoxemia

**Fig 11:**
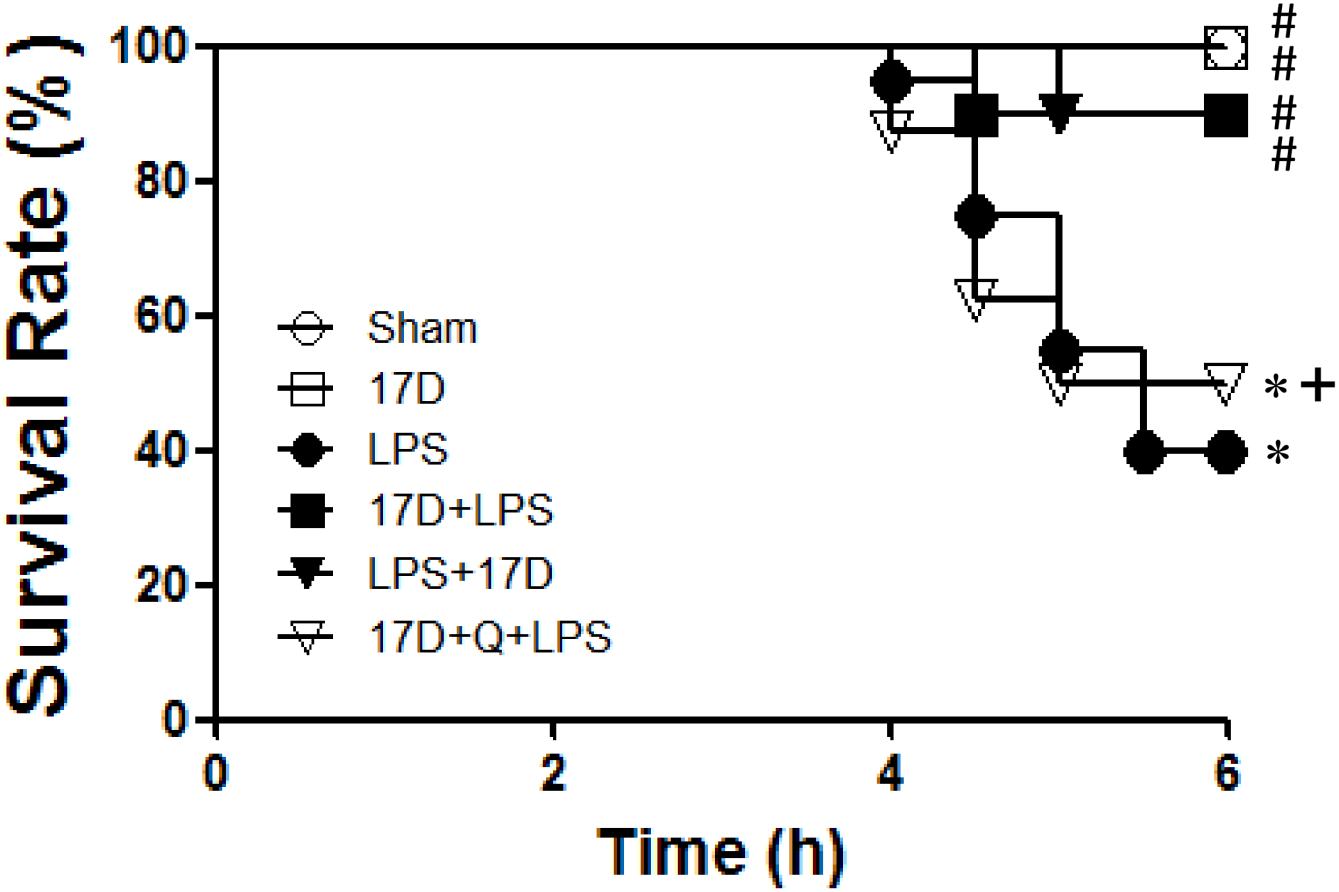
Effects of 17-DMAG on survival rate of rats after LPS administration. 17-DMAG (17D; 5 mg/kg) was given 20 h or 0 min prior to LPS initiation (30 mg/kg/4 h). Quercetin (Q; 300 mg/kg, i.p.), an HSP70 inhibitor, was given 6 h prior to LPS initiation. Data are shown as mean± SEM. *P < 0.05 vs. the sham group; ^#^P < 0.05 vs. the LPS group, ^+^P < 0.05 vs.17D+LPS group; n = 5–20.

There is no mortality was found in sham and 17-DMAG treated groups, which indicate that all rats were survived (100%) without causing any toxic effects by 17-DMAG. However, the survival rate reduced to 40% in LPS group (8 rats survived and 12 died). Moreover, the 90% (9 rats survived and 1 died) survival rate was observed in 17D+LPS and 17D+LPS (co-treatment) groups which is significantly higher than that of LPS-induced untreated group (P< 0.05). Pretreatment with quercetin, an HSP70 inhibitor, significantly affect the beneficial effect of 17-DMAG on survival rate in endotoxemia (17D+Q+LPS: 4 survived and 4 died, survival rate 50%) (Fig 11).

## Discussion

17-DMAG is a geldanamycin analog, reported to have less hepatic and renal toxicity compared with the original geldanamycin [12]. It has been shown that 17-DMAG attenuates the inflammatory responses in macrophage cells [23], hemorrhage-induced intestinal injury [24], and experimental animal model-induced stroke [25]. 17-DMAG induces HSP70 protein expression via activating HSF-1, an HSP70 transcription factor, and interferes the binding of NF-κB to the TNF-α transcription element to inhibit the TNF-α transcription, leading to amelioration of liver injury caused by LPS [11]. Thus, in the present study, we evaluated the effectiveness of 17-DMAG on septic shock, MODS and survival rate in endotoxemic rats, and explored the role of HSP70. 17-DMAG ameliorated circulatory dysfunction, multiple organ injuries and mortality caused by endotoxemia. The antioxidant and anti-inflammatory effects of 17-DMAG contribute to the protective effects on MODS. Several beneficial effects of 17-DMAG were reversed by quercetin, an HSP70 inhibitor, which is mediated via decreasing the HSP70 protein expression and interfering the HSP70 function [15,16,26,27], suggesting that HSP70 induction plays an important role in the protective effect of 17-DMAG.

In several previous studies, 17-DMAG inhibits NO/iNOS pathway and TNF-α release via inhibiting NF-κB activation in hemorrhage-induced small intestine injury [24]. In gamma-irradiated human T cells, 17-DMAG was reported to inhibit the elevation of iNOS and NF-κB p65 protein expression, resulting in the decreases of NO production, protein nitration, and lipid peroxidation [28]. Another study proposed that 17-DMAG inhibits the release of pro-inflammatory cytokines in LPS-induced septic mice [11]. In the present study, 17-DMAG not only reduced the releases of LPS challenged NO, IL-6, and TNF-α, but also suppressed iNOS expression in hearts, livers and lungs. Moreover, 17-DMAG suppressed the increase in NF-κB pp65 levels caused by LPS in lungs. These evidences indicate that 17-DMAG possesses powerful anti-inflammatory effects on sepsis, which is likely mediated by suppression of NF-κB activation. Most of these effects were reversed by quercetin implying that the anti-inflammatory effects of 17-DMAG is associated with induction of HSP70. However, quercetin is not a specific inhibitor of HSP70. It has been shown that quercetin is a pleiotropic kinase inhibitor [29]. The ability of quercetin to reverse the beneficial effect of 17-DMAG on LPS-induced damage may be through a multitude of mechanisms, including inhibition of HSP70.

17-DMAG reduced oxidative stress caused by LPS, evidenced by attenuation of superoxide anions production and elevation of GSH in hearts. However, these actions were not reversed by quercetin (Fig 5). It has been reported that quercetin can exert antioxidant action to show protective effects [29]. This may explain why quercetin did not reverse the anti-oxidative stress effect of 17-DMAG. Recombinant human HSP70 has been reported to reduce lipoteichoic acid-induced ROS production in human neutrophils and monocytes [8]. Furthermore, 17-DMAG diminished the expression of Nox1, a superoxide-generating NADPH oxidase, and Nox organizer-1 (Noxo1) in VSMCs and monocytes [30]. Accordingly, induction of HSP70 by 17-DMAG might contribute to suppress oxidative stress during sepsis. In the present study, 17-DMAG significantly induced and maintained a high level of HO-1, an antioxidant protein [31], till 6 h after LPS initiation (Fig 9). Therefore, 17-DMAG may exert an antioxidant action via induction of HO-1, an HSP70-independent route.

The cardiac caspase 3 activation induced by LPS was significantly inhibited by 17-DMAG, which was reversed by quercetin (Fig 3), demonstrating that 17-DMAG possesses anti-apoptotic effect and the inhibitory effect on caspase 3 activity is HSP70-dependent. It has been shown that HSP70 exerts anti-apoptotic activity by blocking the recruitment of procaspase-9 to the Apaf-1/dATP/cytochrome c apoptosome complex, causing the caspase 3 activity inhibition [24,28]. In this study, 17-DMAG induced an increase in HSP70 expression during sepsis, which may participate in suppression of caspase 3 activation. This anti-apoptotic effect contributes to the protective effect of 17-DMAG on severe sepsis.

Sepsis-induced procoagulation is mainly due to the release of cytokines by endotoxins, which in turn triggers the expression of tissue factor on endothelial cells and monocytes [32]. The plasma level of IL-6 is suggested as a coagulation marker [33,34]. Several studies reported that IL-6 is the most important mediator in coagulation activation, because it can increase the expression of tissue factor on monocytes and endothelial cells [35,36]. 17-DMAG reduced the prolongation of prothrombin time, ameliorated the reduction of platelet count, and suppressed the elevation of PAI-1 levels by LPS, indicating the coagulation function improved, which may be mediated by suppression of IL-6 and TNF-α release (Fig 4B and 4C). Amelioration of DIC by 17-DMAG can prevent decreased blood perfusion, inadequate oxygenation and multiple organ dysfunction or failure during sepsis, leading to improving survival rate.

Playing as a molecular chaperone, HSP90 protein regulates several intracellular signal transduction pathways and cell fate pathway [37,38]. Common client proteins of HSP90 chaperone are protein kinases. HSP90 regulates their biogenesis, stability, and enzymatic activity [39]. It has been reported that HSP90 inhibitor geldanamycin inhibits the function of the IκB kinase signalosome, resulting in suppression of the activation of NF-κB [40,41]. Inhibition of HSP90 by geldanamycin analogue 17-allylamino-17-demethoxy-geldanamycin (17-AAG) attenuates LPS-induced increase in retinal NF-κB activity, leading to suppression of inflammation in LPS-induced uveitis [42]. Additionally, geldanamycin decreases CD14 surface expression in murine macrophages to ameliorate the response to LPS [43]. 17-AAG suppresses the LPS-induced increase in membranous CD14 expression of leukocytes in rats [42]. The other HSP90 inhibitor AT13387 reduces the expression of Toll-like receptor 4 (TLR4) and inflammatory chemokines in the kidney following renal ischemia-reperfusion injury [44]. Because CD14 is a co-receptor for LPS binding to TLR4, modulation of CD14 and TLR4 by HSP90 inhibition was a possible mechanism for the decrease in the LPS response. The LPS-induced activation of TLR4 signaling to NF-κB can be targeted by HSP90 inhibitors. Moreover, HSP90 has been identified to be an important endogenous protein enhancer of iNOS to facilitate NO synthesis and cytotoxicity [45]. It also plays an obligatory role in iNOS induction [46] and increases iNOS activity by driving heme insertion in an ATP-dependent process [47]. Therefore, besides induction HSP70, direct inhibition of HSP90 by 17-DMAG, resulting in suppression of TLR4 expression, NF-κB activation, and iNOS activity, contributes to the beneficial effect on MODS caused by endotoxemia.

In conclusion, 17-DMAG prevents hypotension, reduces MODS and increases survival rate in severe sepsis. Induction of HSP70 and HO-1 by 17-DMAG may result in the antioxidant and anti-inflammatory effects, which contributes to the protective effect of 17-DMAG on sepsis. Overall, these findings suggest that 17-DMAG may consider as a novel therapeutic agent for the prevention of endotoxemia-induced MODS and other inflammatory diseases.
